# Supplementary material for: An RFC4/Notch1 signaling feedback loop promotes NSCLC metastasis and stemness
Source: Nat Commun. 2021 May 11;12:2693. doi: 10.1038/s41467-021-22971-x (PMC8113560; doi:10.1038/s41467-021-22971-x)
Supplement: Supplementary file 1 — Supplementary Information [file 41467_2021_22971_MOESM1_ESM.pdf]

Supplementary Information

An RFC4/Notch1 signaling feedback loop promotes NSCLC metastasis and stemness

Liu, et al.

Supplementary Figure

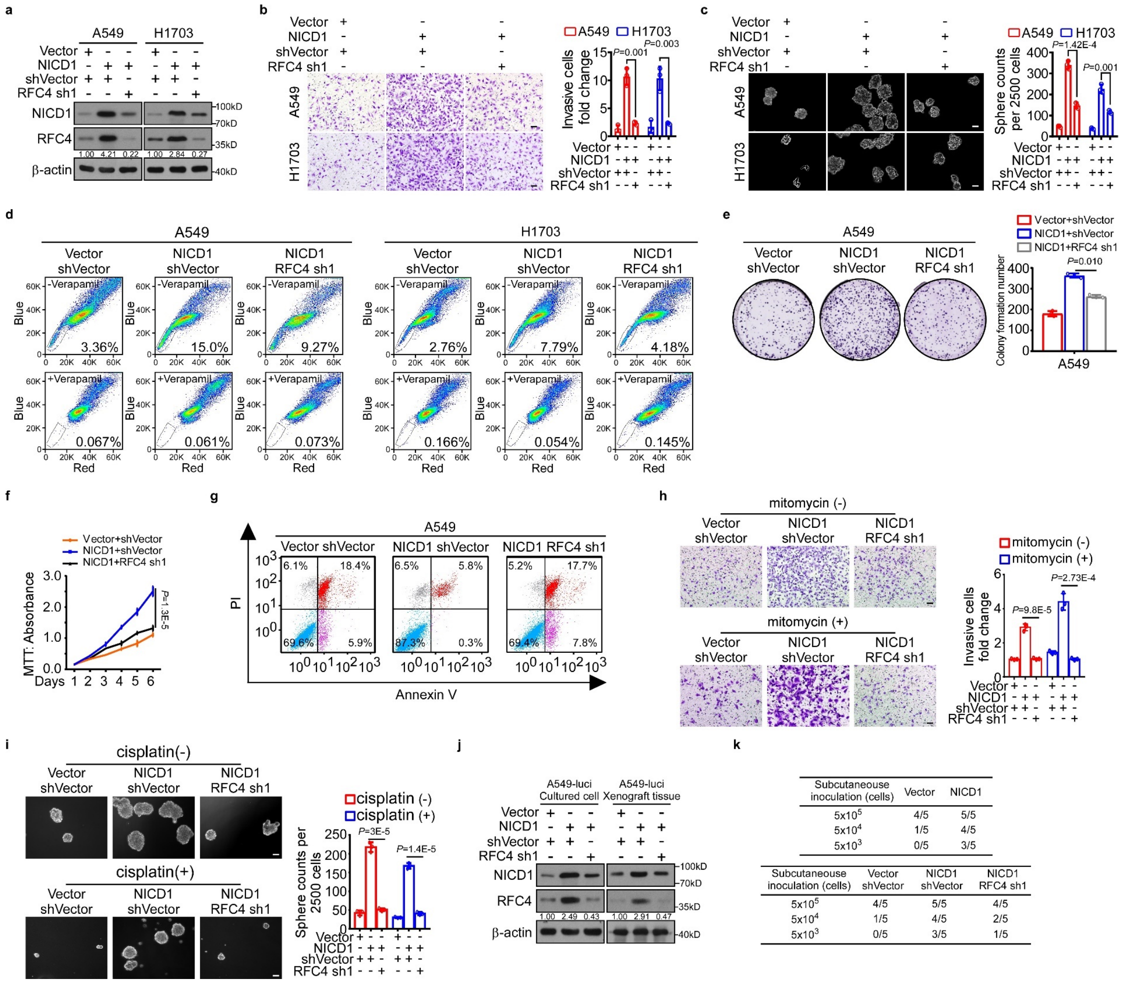

Supplementary Figure 1. RFC4 is essential for Notch activation-induced metastasis and stemness of NSCLC. a The effect of overexpression of NICD1 together with silencing RFC4 on protein levels of NICD1 and RFC4 in A549 and H1703 cells. Representative images of three independent reproducible experiments are shown. **b** and **c** Representative images of three independent reproducible experiments and quantitation of cells invasion or tumor spheres formation by the indicated cells. Scale bar: 50 µm. **d** The effect of overexpressing NICD1 or together with RFC4 silencing on the proportion of side-population (SP) cells as evaluated by flow cytometry analysis. Indicated cells were subjected to Hoechst 33342 dye staining, which is detected at two emission wavelengths after 350 nm excitation: Hoechst Red (675 nm) and Hoechst Blue (450 nm), and cells that actively efflux the Hoechst dye appear as a distinct population were known as the side-population. Verapamil treatment was

used as the negative control. **e** Representative images of three independent reproducible experiments and quantitation of cells colony formation by NICD1 or together with RFC4 silencing in A549. **f** MTT assay revealed cell growth curves of NICD1 or together with RFC4 silencing in A549 cells. **g** Flow cytometry analysis of cell apoptosis for NICD1 or together with RFC4 silencing in A549 cells. Highly fluorescent Annexin V-FITC indicated cells with apoptosis and highly fluorescent Propidium Iodide (PI) indicated cell with necrosis. **h** The effect of overexpressing NICD1 or together with RFC4 silencing on cells invading through matrigel, without (-) or with (+) Mitomycin treatment. Representative images of three independent reproducible experiments are shown. Scare bar: 50 µm. **i** The effect of overexpressing NICD1 or together with RFC4 silencing on tumor spheres formation, without (-) or with (+) cisplatin treatment. Representative images of three independent reproducible experiments are shown. Scare bar: 50 µm. **j** The effect of overexpression of NICD1 together with silencing RFC4 on protein levels of NICD1 and RFC4 in A549-luci cultured cell and xenograft tissues. Representative images of three independent reproducible experiments are shown. **k** Tumor xenografts of the indicated cells subcutaneously implanted with different cell numbers and tumor formation frequencies are shown. Data in panel **b**, **c**, **e**, **f**, **h** and **i** are presented as mean ± SD derived from three independent experiments. Two-way ANOVA multiple comparison analysis was used for statistical analysis. Source data are provided as a Source Data file.

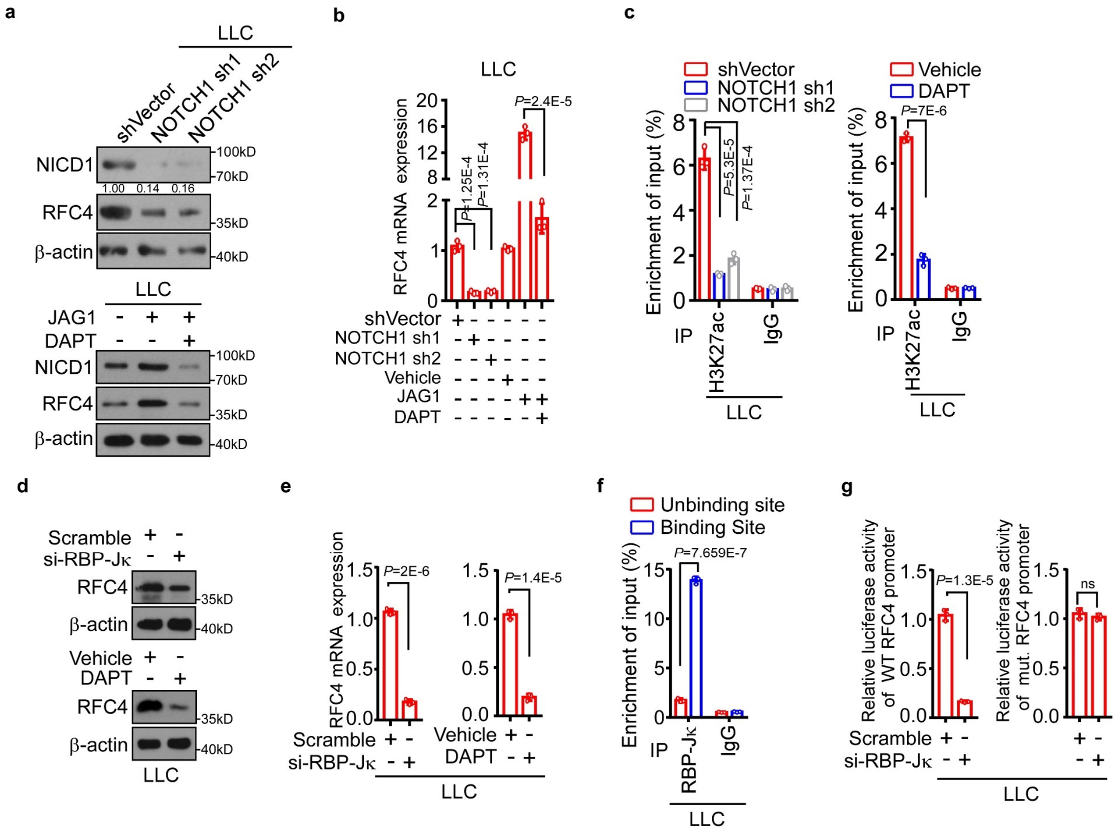

Supplementary Figure 2. RFC4 is a *de novo* direct transcriptional target of Notch1 signaling. a and **b** The protein and mRNA levels of RFC4 in LLC cells by activating Notch signaling by JAG1 or inhibiting Notch signaling by treatment of silencing Notch1 or a γ-secretase inhibitor DAPT. Representative images of three independent reproducible experiments are shown. **c** ChIP analysis following H3K27ac immunoprecipitation shows the interaction between H3K27ac and the promoter region of the RFC4 gene in response to silence of NOTCH1 or treatment of DAPT. IgG immunoprecipitation was used as a negative control. **d** and **e** The both protein and mRNA levels of RFC4 by silencing RBP-Jκ or treating with DAPT in LLC cells. Representative images of three independent reproducible experiments are shown. **f** ChIP enrichment assay shows binding of RBP-Jκ to the predicted binding site in the promoter region of *RFC4* in LLC cells. IgG immunoprecipitation was used as a negative control. **g** The effects of RBP-Jκ depletion on luciferase activities of the reporter constructs spanning wildtype or mutant predicted putative binding site for RBP-Jκ in the promoter region of *RFC4*. Data in panel **b**, **c** and **e**-**g** are presented as mean ± SD derived from three independent experiments. Two-way ANOVA multiple

comparison analysis was used for statistical analysis. ns, not significant. Source data are provided as a Source Data file.

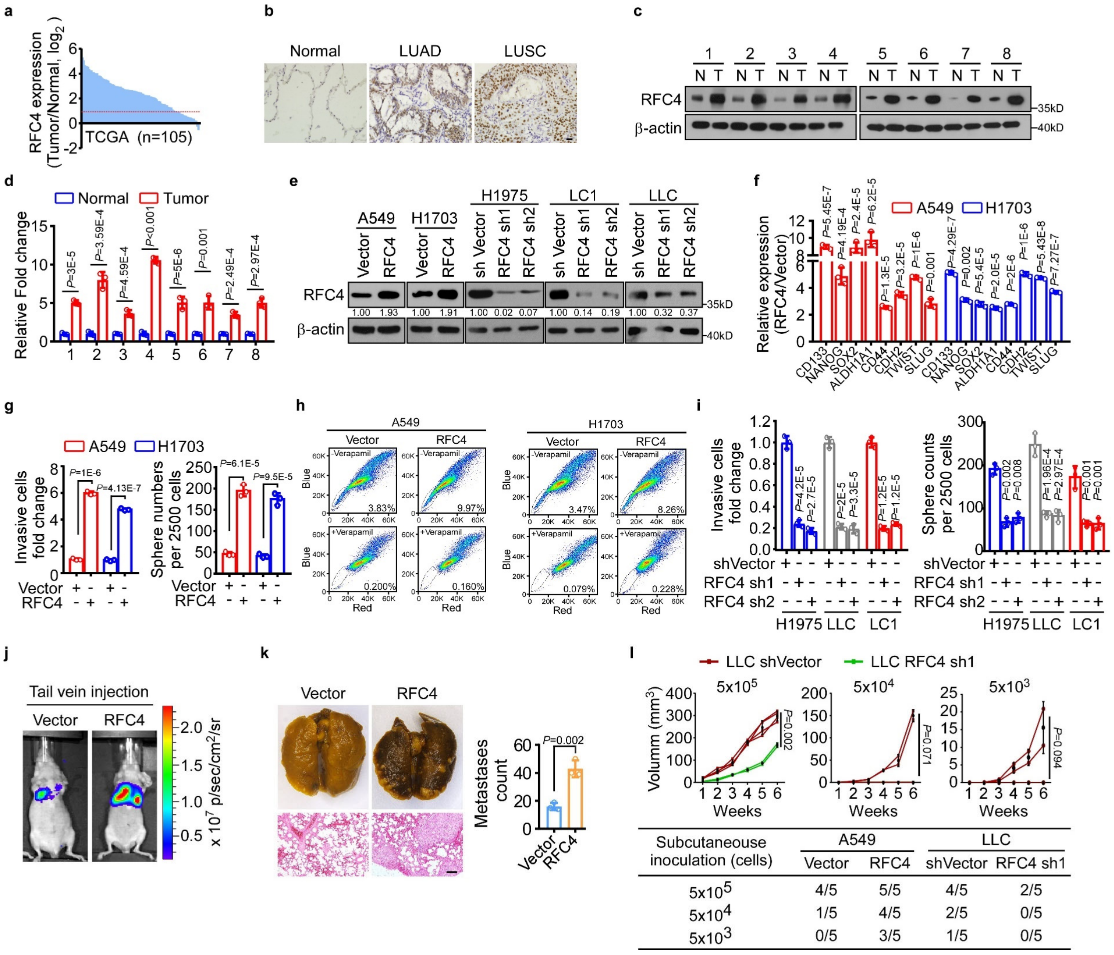

Subcutaneous inoculation (cells)	A549		LLC	
	Vector	RFC4	shVector	RFC4 sh1
$5x10^5$	4/5	5/5	4/5	2/5
$5x10^4$	1/5	4/5	2/5	0/5
$5x10^3$	0/5	3/5	1/5	0/5

Supplementary Figure 3. RFC4 promotes NSCLC metastasis and stemness both *in vitro and in vivo*. a Analysis of the TCGA lung cancer datasets shows RFC4 expression in 105 cases of NSCLC tissues as normalized to corresponding paired adjacent non-cancerous lung tissues. **b** IHC staining of RFC4 expression in non-cancerous lung tissue, lung adenocarcinoma (LUAD), and lung squamous cell carcinoma (LUSC) tissues of NSCLC patients. Representative images of our 219 NSCLC patients. Scale bar: 20 µm. **c** Expression of RFC4 proteins in 8 pairs of lung cancer (T) tissues and corresponding adjacent normal lung tissues (N). Representative images of three independent reproducible experiments are shown. **d** qRT-PCR analysis of RFC4 mRNA levels in 8 pairs of NSCLC tissue and the corresponding adjacent non-cancerous lung tissue. **e** A549 and H1703 cells stably overexpressing RFC4, and silencing in H1975, LC1 and LLC cells, protein levels of RFC4 were constructed as confirmed by WB analysis. Representative images of three independent reproducible experiments are shown. **f** The effect of overexpressing RFC4 on expression of invasion- or stemness-

associated genes in A549 and H1703 cells. **g** Quantification of invading cells and tumor spheres formed by A549 and H1703 cells overexpressing RFC4, or expressing corresponding vector controls. **h** Flow cytometry analysis of side population (SP) in RFC4 overexpressing A549 and H1703 cells without (-) or with (+) Verapamil treatment (the negative control). Indicated cells were subjected to Hoechst 33342 dye staining, which is detected at two emission wavelengths after 350 nm excitation: Hoechst Red (675 nm) and Hoechst Blue (450 nm), and cells that actively efflux the Hoechst dye appear as a distinct population were known as the side-population. **i** Quantification of invading cells and tumor spheres formed by H1975, LLC, and LC1 cells silenced RFC4, or corresponding vector controls. **j** and **k** Nude mice (n = 5 per group) were intravenously injected with RFC4-overexpressing A549-luci cells. Representative bioluminescent images (**j**), picric acid staining, H&E staining and the numbers of metastatic foci of the indicated lung tissue are shown (**k**). Scale bar: 100 µm. **l** Growth curves of tumor xenografts of RFC4-silenced LLC cells subcutaneously implanted with different cell numbers and tumor formation frequencies for indicated cell numbers are shown, and tumor formation rate of xenografts of RFC4 overexpressing A549 cell or RFC4-silenced LLC cells. Data in panel **d**, **f**, **g**, **i**, **k**, and **l** are presented as mean ± SD derived from three independent experiments. Two-way ANOVA multiple comparison analysis was used for statistical analysis. Source data are provided as a Source Data file.

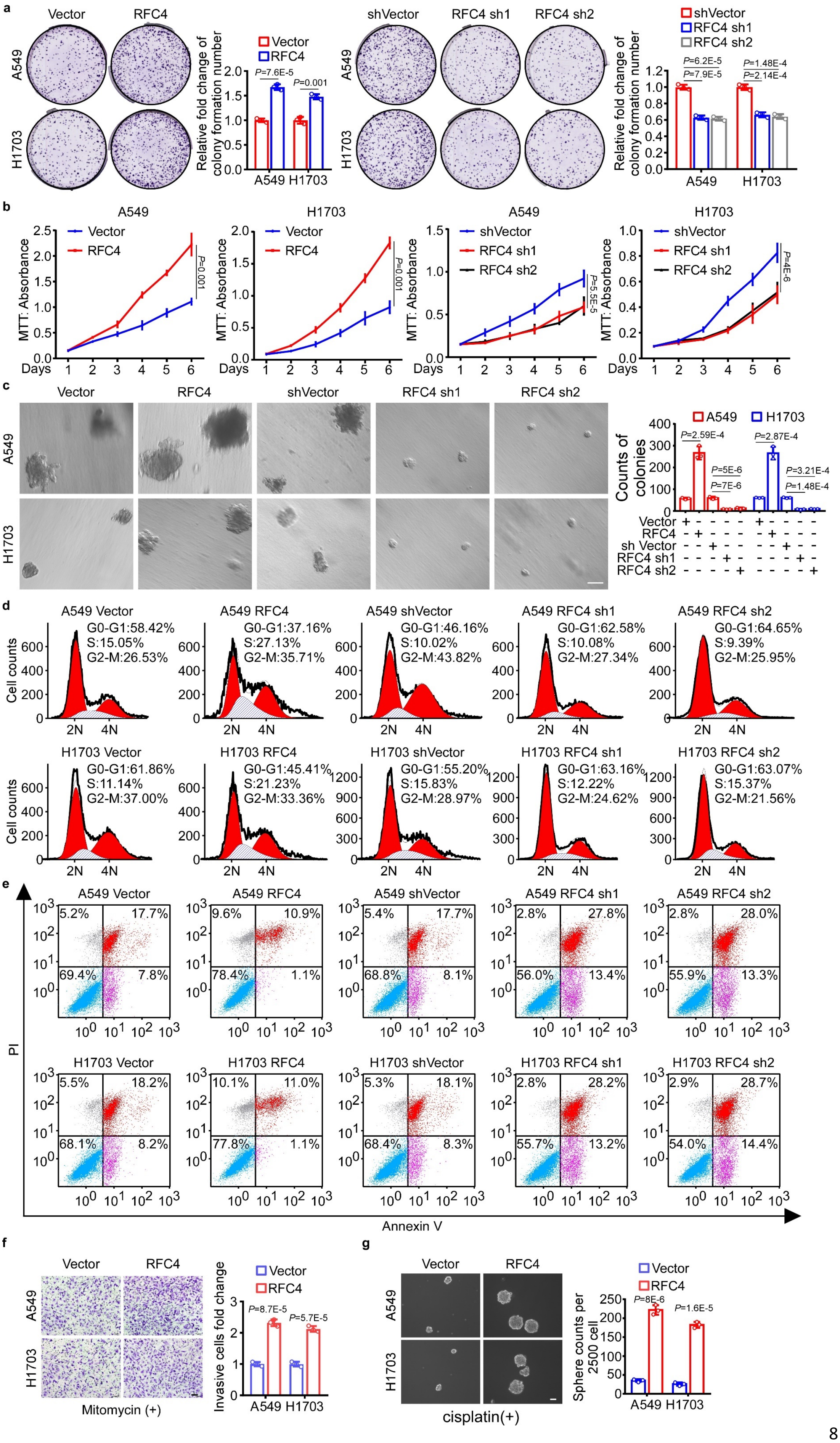
a
Vector
RFC4
A549
H1703
Relative fold change of colony formation number
P=7.6E-5
P=0.001
shVector
RFC4 sh1
RFC4 sh2
P=6.2E-5
P=7.9E-5
P=1.48E-4
P=2.14E-4
b
MTT: Absorbance
Days
P=0.001
P=5.5E-5
P=4E-6
c
Counts of colonies
P=2.59E-4
P=2.87E-4
P=5E-6
P=7E-6
P=3.21E-4
P=1.48E-4
sh Vector
d
Cell counts
A549 Vector G0-G1:58.42% S:15.05% G2-M:26.53%
A549 RFC4 G0-G1:37.16% S:27.13% G2-M:35.71%
A549 shVector G0-G1:46.16% S:10.02% G2-M:43.82%
A549 RFC4 sh1 G0-G1:62.58% S:10.08% G2-M:27.34%
A549 RFC4 sh2 G0-G1:64.65% S:9.39% G2-M:25.95%
H1703 Vector G0-G1:61.86% S:11.14% G2-M:37.00%
H1703 RFC4 G0-G1:45.41% S:21.23% G2-M:33.36%
H1703 shVector G0-G1:55.20% S:15.83% G2-M:28.97%
H1703 RFC4 sh1 G0-G1:63.16% S:12.22% G2-M:24.62%
H1703 RFC4 sh2 G0-G1:63.07% S:15.37% G2-M:21.56%
2N
4N
e
PI
Annexin V
A549 Vector 5.2% 17.7% 69.4% 7.8%
A549 RFC4 9.6% 10.9% 78.4% 1.1%
A549 shVector 5.4% 17.7% 68.8% 8.1%
A549 RFC4 sh1 2.8% 27.8% 56.0% 13.4%
A549 RFC4 sh2 2.8% 28.0% 55.9% 13.3%
H1703 Vector 5.5% 18.2% 68.1% 8.2%
H1703 RFC4 10.1% 11.0% 77.8% 1.1%
H1703 shVector 5.3% 18.1% 68.4% 8.3%
H1703 RFC4 sh1 2.8% 28.2% 55.7% 13.2%
H1703 RFC4 sh2 2.9% 28.7% 54.0% 14.4%
f
Mitomycin (+)
Invasive cells fold change
P=8.7E-5
P=5.7E-5
g
cisplatin(+)
Sphere counts per 2500 cell
P=8E-6
P=1.6E-5

Supplementary Figure 4. RFC4 promotes NSCLC cell growth. a Representative images and quantitation of cells colony formation by RFC4 overexpression or silencing in A549 and H1703 cells. **b** MTT assay revealed cell growth curves of RFC4 overexpression or silencing in A549 and H1703 cells. **c** Representative images and quantitation of cell colonies of RFC4 overexpression or silencing in A549 and H1703 cells grown in the three dimensional soft-agar assays. Scale bar: 50 μm. **d** Cell cycle analysis through PI staining and following flow cytometry for RFC4 overexpression or silencing in A549 and H1703 cells. **e** Flow cytometry analysis of cell apoptosis for RFC4 overexpression or silencing in A549 and H1703 cells. Highly fluorescent Annexin V-FITC indicated cells with apoptosis and highly fluorescent Propidium Iodide (PI) indicated cell with necrosis. **f** Representative images of three independent reproducible experiments and quantitation of tumor cells invading through matrigel for RFC4 overexpression A549 and H1703 cells. Scale bar: 50 μm. **g** The effect of overexpressing RFC4 on tumor spheres formation, without (-) or with (+) cisplatin treatment in A549 and H1703 cells. Representative images of three independent reproducible experiments are shown. Scare bar: 50 μm. Data in panel **a**, **b** and **f** are presented as mean ± SD derived from three independent experiments. Two-way ANOVA multiple comparison analysis were used for statistical analysis. Source data are provided as a Source Data file.

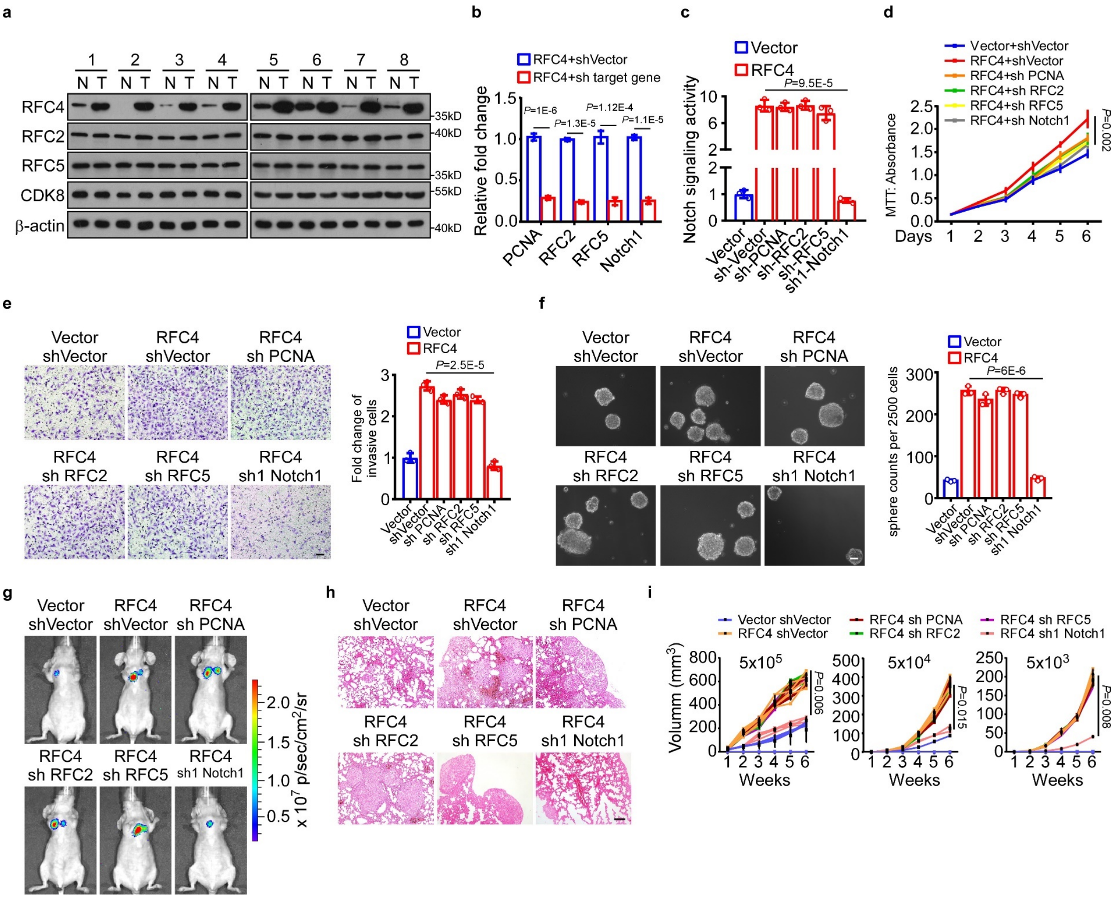

Supplemental Figure 5. RFC4 promotes metastasis and stemness that is independent on the replication factor C complex. a The protein levels of RFC4, RFC2, RFC5 and CDK8 in 8 pairs of NSCLC tissues and adjacent non-cancerous lung tissues. Representative images of three independent reproducible experiments are shown. **b** Relative mRNA folds change of target genes in RFC4 or together with silencing genes in A549 cells. **c** The effect of activating Notch signaling by overexpression RFC4 or together with silencing PCNA, RFC2, RFC5 or Notch1. **d** MTT assay revealed cell growth curves of RFC4 overexpression or together with silencing PCNA, RFC2, RFC5 or Notch1. **e** Representative images and quantitation of tumor cells invading through matrigel for RFC4 overexpression or together with silencing PCNA, RFC2, RFC5 or Notch1 in A549 cells. Scale bar: 50 μm. **f** The effect of overexpressing RFC4 or together with silencing PCNA, RFC2, RFC5 or Notch1 on tumor spheres formation. Scale bar: 50 μm. **g** and **h** Nude mice (n = 5 per group) were intravenously injected with indicated cells. Bioluminescent images (**g**) and H&E staining (**h**) of the indicated lung tissue are shown. Six representative cases are shown. Scale bar: 100 μm. **i**

Growth curves of tumor xenografts of the indicated cells subcutaneously implanted with different cell numbers and tumor formation frequencies for indicated cell numbers are shown. Data in panel **b**-**e** and **i** are presented as mean ± SD derived from three independent experiments. Two-way ANOVA multiple comparison analysis was used for statistical analysis. Source data are provided as a Source Data file.

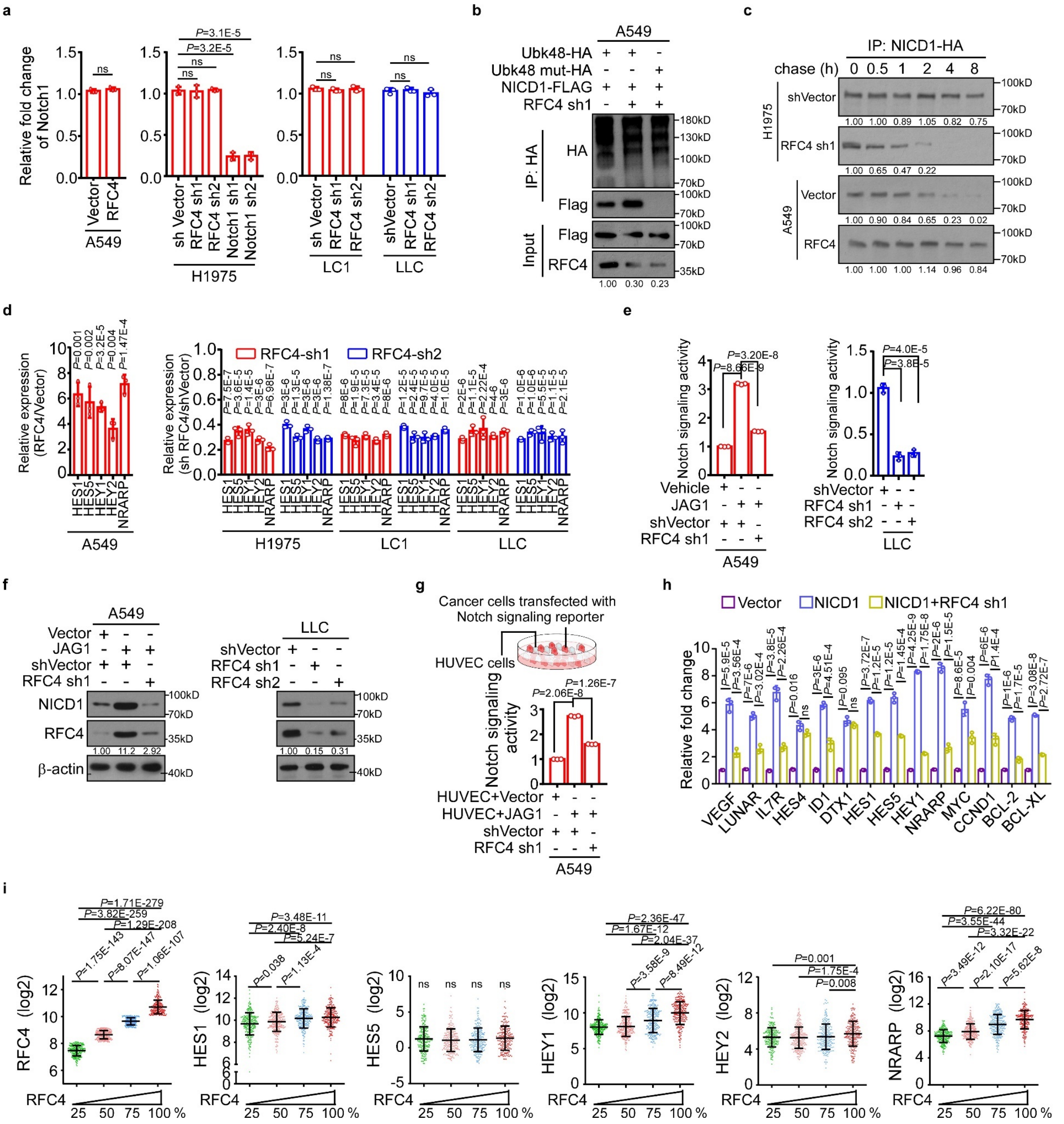

Supplementary Figure 6. RFC4 promotes NICD1 protein stability to form a positive feedback loop. a qRT-PCR analysis of Notch1 level in RFC4 overexpressing A549 cells, RFC4-silenced H1975, LC1, and LLC cells, and Notch1-silenced H1975 cells. **b** The effect of silencing RFC4 on the levels of K48-linked polyubiquitination of NICD1 was evaluated by immunoprecipitation of HA-tagged ubiquitin in A549 cells. A dominant-negative mutant form of HA-tagged ubiquitin (UbK48R-HA) was used as a negative control. Representative images of three independent reproducible experiments are shown. **c** Pulse-chase analysis showed the effect of silenced or overexpressing RFC4 on the half-lives of NICD1 in H1975 and A549 cells. Representative images of three independent reproducible experiments are shown. **d** The effect of overexpressing

RFC4 in A549 cells or silencing RFC4 expression in H1975, LC1 and LLC on mRNA expression of the indicated genes. **e and f** The reversing effect of silencing RFC4 on Notch signaling activities in A549 cells treated with JAG1 or LLC cells. Representative images of three independent reproducible experiments are shown. **g** NSCLC cells silenced with RFC4 were transfected with the Notch1-driven luciferase reporter and co-cultured with HUVEC cells overexpressing JAG1 or vector controls. Dual-luciferase assays revealed Notch signaling activities. **h** The effect of silencing RFC4 on expression of the indicated mRNAs in NICD1-overexpressing cells. **i** Expression of RFC4 and HES1, HES5, HEY1, HEY2 and NRARP levels of 971 NSCLC patients in TCGA lung cancer datasets grouped according to RFC4 expression. Data in panel **a** and **d**-**i** are presented as mean ± SD derived from three independent experiments. Two-way ANOVA multiple comparison analysis and two-tailed unpaired Student's *t* test were used for statistical analysis. Source data are provided as a Source Data file.

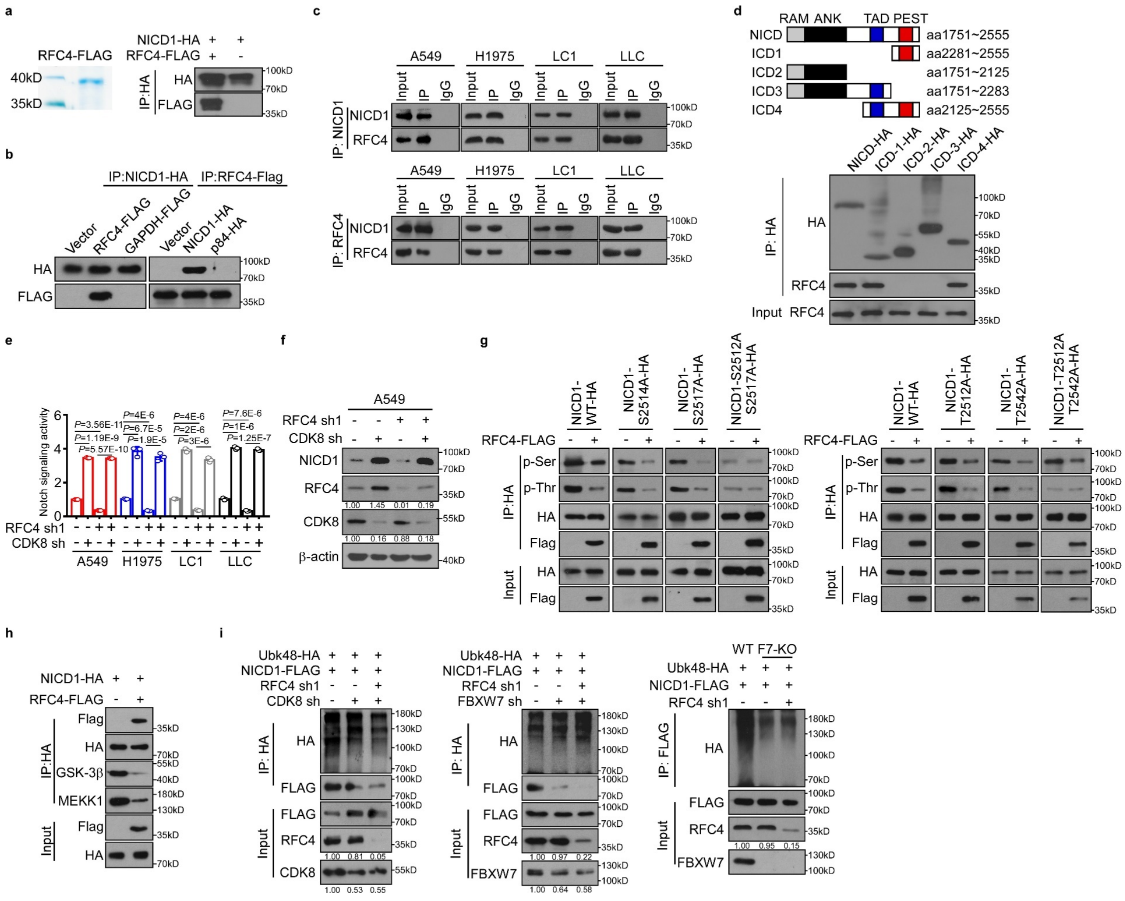

Supplementary Figure 7. RFC4 binds to stabilize NICD1 by abrogating CDK8/FBXW7-induced degradation. a Recombinant RFC4-FLAG proteins were incubated with NICD1-HA-associated affinity gels, eluted and subjected to WB analysis. **b** Immunoprecipitation assay revealing interaction between RFC4 with NICD1, GAPDH-FLAG and p84-HA immunoprecipitation was used as a negative control. **c** The interaction between NICD1 and RFC4 in the presence of RFC4 or Notch1 silencing was evaluated by immunoprecipitation of RFC4 or Notch1 in A549 and H1975 cells. **d** Schematic representation of C-terminal HA-tagged NICD1, along with indicated truncated constructions. 293FT cells were co-transfected with RFC4-FLAG along with the indicated truncated NICD1-HA constructions, and the interactions between RFC4 and NICD1 were determined by immunoprecipitations. **e** and **f** The reversing effects of silencing RFC4 or CDK8 on Notch signaling activities and NICD1 protein levels. **g** WB analysis of the effect of overexpression RFC4 on serine and threonine phosphorylation levels of wildtype or mutated NICD1 by HA-tagged NICD1 immunoprecipitation. **h** The interaction between NICD1 and GSK-3β and MEKK1 in

the presence or absence of RFC4-FLAG was evaluated by immunoprecipitation of NICD1-HA. **i** The effect of silencing RFC4 or together with CDK8 or FBXW7 silencing on the levels of K48-linked polyubiquitination of NICD1 was evaluated by immunoprecipitation of HA-tagged ubiquitin in A549 or A549 FBXW7 knockout cell. Representative images of three independent reproducible experiments are shown (**a**-**d**, **f**-**i**). Data in panel **e** is presented as mean ± SD derived from three independent experiments. Two-way ANOVA multiple comparison analysis was used for statistical analysis. Source data are provided as a Source Data file.

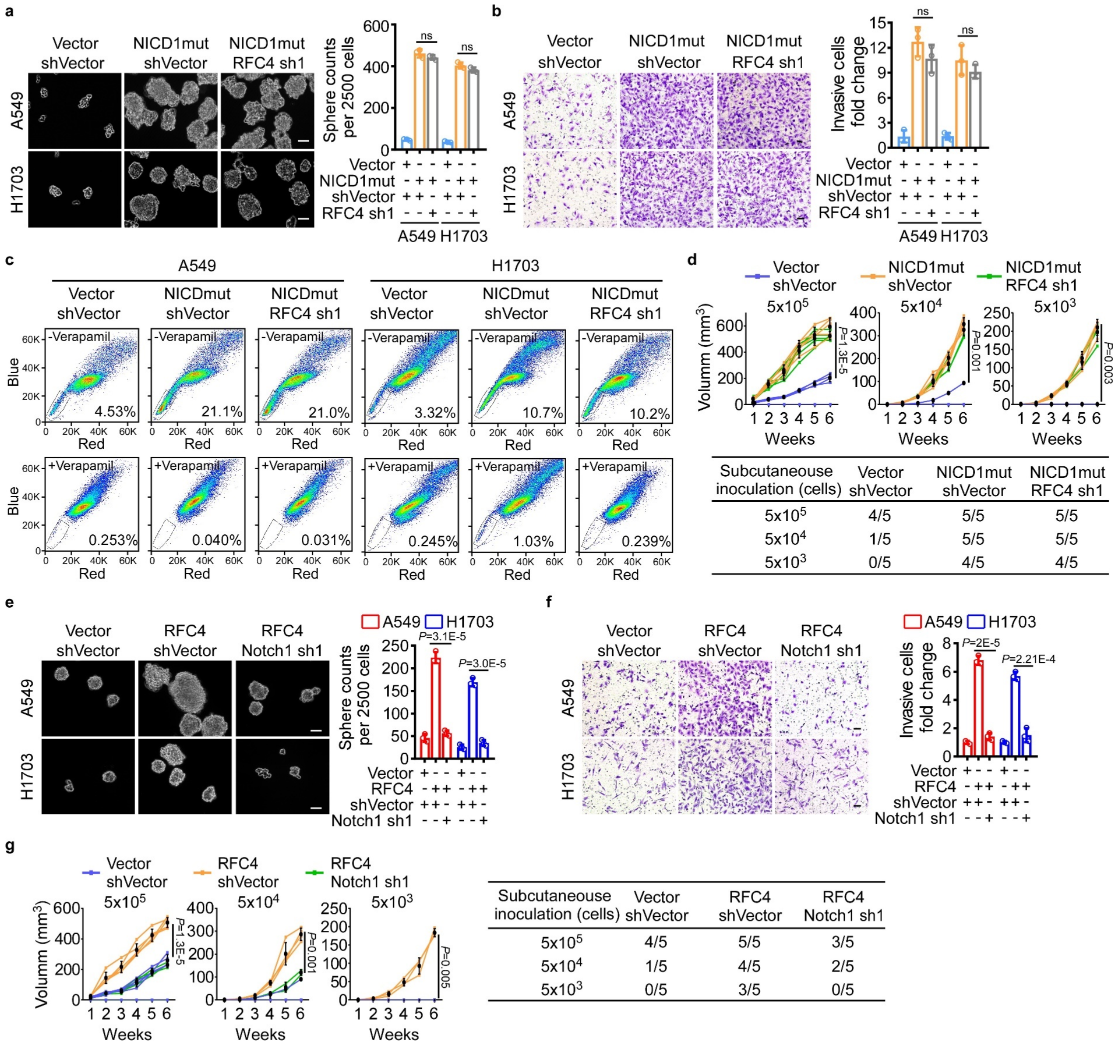

Supplementary Figure 8. RFC4-induced stabilization of NICD1 promotes NSCLC aggressiveness and resists treatment with γ-secretase inhibitor. a and **b** Representative images and quantitation of cells tumor spheres formed or invading through matrigel by A549 and H1703 cells overexpressing stabilized NICD1 or together with RFC4 silencing. Scale bar: 50 µm. **c** Flow cytometry analysis of side population (SP) in the indicated cells without (-) or with (+) Verapamil treatment (the negative control). Indicated cells were subjected to Hoechst 33342 dye staining, which is detected at two emission wavelengths after 350 nm excitation: Hoechst Red (675 nm) and Hoechst Blue (450 nm), and cells that actively efflux the Hoechst dye appear as a distinct population were known as the side-population. **d** Growth curves of tumor xenografts of the indicated cells subcutaneously implanted with different cell numbers and tumor formation frequencies for indicated cell numbers are shown. **e** and **f** Representative images and quantitation of cells tumor spheres formed or

invading through matrigel by A549 and H1703 cells overexpressing RFC4 or together with Notch1 silencing. Scale bar: 50 µm. **g** Growth curves of tumor xenografts of the indicated cells subcutaneously implanted with different cell numbers and tumor formation frequencies for indicated cell numbers are shown. Data in panel **a**, **b** and **d**-**g** are presented as mean ± SD derived from three independent experiments. Two-way ANOVA multiple comparison analysis was used for statistical analysis. Source data are provided as a Source Data file.

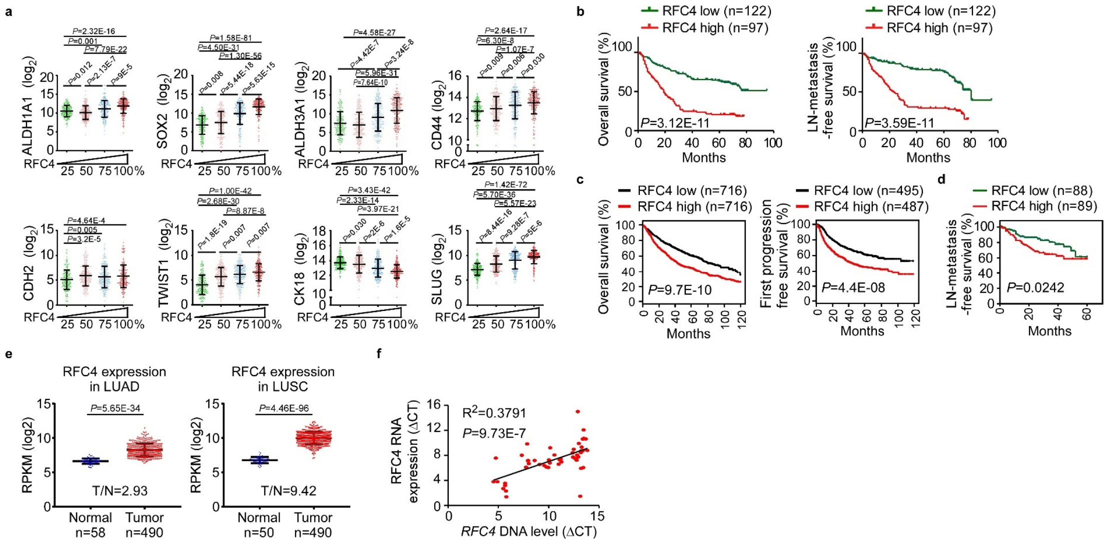

Supplementary Figure 9. RFC4 is amplified in NSCLC and correlates with NSCLC progression. a Expression of metastasis- and stemness-associated genes, including ALDH1A1, SOX2, ALDH3A1, CD44, CDH2, TWIST1, CK18 and SLUG, in the 971 NSCLC patients in the TCGA lung cancer datasets grouped according to RFC4 expression, was compared. **b-d** Kaplan-Meier analysis of the correlation between RFC4 expression and the indicated prognosis of NSCLC patients from our collected cohort (**b**), the online kmplot database (**c**) and MSKCC dataset (**d**). The medians of each genes' expression were used as the cut-off value. **e** Analysis of RFC4 expression in human LUAD and LUSC tissue (Tumor) and adjacent non-tumor tissue (Normal). **f** The correlation between mRNA and DNA levels of *RFC4* is shown. Data in panel **a** and **e** are presented as mean ± SD derived from three independent experiments. Two-way ANOVA multiple comparison analysis and two-tailed unpaired Student's *t* test were used for statistical analysis. Source data are provided as a Source Data file.

Supplementary Table 1. Clinicopathologic characteristics of 219 NSCLC patients

Characteristics		No of cases (%)
**Age (y)**	≤60	133 (60.7)
	>60	86 (39.3)
**Gender**	male	163 (74.4)
	female	56 (25.6)
**Pathologic type**	Squamous cell carcinoma	74 (33.8)
	Adenocarcinoma	90 (41.1)
	Adenosquamous carcinoma	17 (7.8)
	Others	38 (17.4)
**Clinical stage**	I	84 (38.4)
	II	50 (22.8)
	III	67 (30.6)
	IV	18 (8.2)
**T classification**	T1	36 (16.4)
	T2	117 (53.4)
	T3	54 (24.7)
	T4	12 (5.5)
**N classification**	N0	116 (53.0)
	N1	53 (24.2)
	N2	48 (21.9)
	N3	2 (0.9)
**Distant metastasis**	M0	201 (91.8)
	M1	18 (8.2)

Supplementary Table 2. Correlation between the clinical pathologic feature and expression of RFC4 in 219 NSCLC patients

Characteristics		RFC4		*p*-value
		High	Low	
Gender	male	75	88	0.382
	female	22	34	
Age (y)	>60	40	46	0.595
	≤60	57	76	
Pathologic type	Squamous cell carcinoma	31	43	0.604
	Adenocarcinoma	44	46	
	Adenosquamous carcinoma	8	9	
	Others	14	24	
Clinical staging	I	16	68	1.45E-9
	II	22	28	
	III	45	22	
	IV	14	4	
T	T1	8	28	5.28E-4
	T2	47	70	
	T3	35	19	
	T4	6	5	
N	N0	37	79	3.2E-5
	N1	24	29	
	N2	34	14	
	N3	2	0	
M	M0	83	118	0.003
	M1	14	4	

Two-tailed Chi-Square test

Supplementary Table 3. Oligos used for knockdown or knockout genes

Name	Sequence (5'-3')
Human RFC4 sh1	GACGTACCATGGAGAAGGA
Human RFC4 sh2	GACCAAGGATCGAGGAGTA
Mouse Rfc4 sh1	CCTGAACTCTTTCGATTAA
Mouse Rfc4 sh2	GGAGCGATTACTGGATATT
Human Notch1 sh1	GATGCGAGATCGACGTCAA
Human Notch1 sh2	GACGGACCCAACACTTACA
Human CDK8 sh	GGAGCAAGGCATTATACCA
Mouse Cdk8 sh	GTACCGAGCTCCAGAATTA
Human FBXW7 sh	GCGTTGTATGCATCTTCAT
Mouse Fbxw7 sh	GAATGGAACTCAAAGACAA
PCNA sh	GGAGAAAGTTTCAGACTAT
RFC2 sh	AATGTGCCCAACATCATCAT
RFC5 sh	CATCATTCGAGGACCGATC
BOLA2B sh	CGGCCAAGTTCGAGGGGAA
PROK1 sh	CCACGCGAGTCTCAATCAT
SLUG sh	CATTAGTGATGAAGAGGAA
KCNA5 sh	GGTTCTCCCGGAACATCAT
HES5 sh	GGAAGCCGGTGGTGGAGAA
TFF1 sh	CCGTGAAAGACAGAATTGT
ANKRD1 sh	TTTCAGAGATGGAGAGTAT
SPRY4 sh	GAGAATGACTACATAGACA
CALCB sh	AGGACTATGTGCAGATGAA
RBP-JK si	CTGGAATACAAGTTGAACA
FBXW7 sg	CTTACCCGTCTTCGACAAAA

Supplementary Table 4. Sense and antisense primers used for qRT-PCR

Name	Sequence (5'-3')
ALDH1A1 sence	GCACGCCAGACTTACCTGTC
ALDH1A1 antisence	CCTCCTCAGTTGCAGGATTAAAG
SOX2 sence	GCCGAGTGGAAACTTTTGTCG
SOX2 antisence	GGCAGCGTGTACTTATCCTTCT
OCT4 sence	CTTGAATCCCGAATGGAAAGGG
OCT4 antisence	GTGTATATCCCAGGGTGATCCTC
TWIST1 sence	GTCCGCAGTCTTACGAGGAG
TWIST1 antisence	GCTTGAGGGTCTGAATCTTGCT
CDH2 sence	TCAGGCGTCTGTAGAGGCTT
CDH2 antisence	ATGCACATCCTTCGATAAGACTG
MYC sence	GGCTCCTGGCAAAAGGTCA
MYC antisence	CTGCGTAGTTGTGCTGATGT
MYCN sence	ACCCGGACGAAGATGACTTCT
MYCN antisence	CAGCTCGTTCTCAAGCAGCAT
CCND1 sence	GCTGCGAAGTGGAAACCATC
CCND1 antisence	CCTCCTTCTGCACACATTTGAA
VEGFA sence	AGGGCAGAATCATCACGAAGT
VEGFA antisence	AGGGTCTCGATTGGATGGCA
BCL2 sence	GGTGGGGTCATGTGTGTGG
BCL2 antisence	CGGTTCAGGTACTCAGTCATCC
NRARP sence	TCAACGTGAACTCGTTCGGG
NRARP antisence	ACTTCGCCTTGGTGATGAGA
Human RFC4 sence	CCGCTGACCAAGGATCGAG
Human RFC4 antisence	AGGGAACGGGTTTGGCTTTC
Mouse RFC4 sence	CAAAGCACAACTGACCAAGGA
Mouse RFC4 antisence	CCAGGTGGCCCATAGAACAAG
Human NOTCH1 sence	GAGGCGTGGCAGACTATGC

Human NOTCH1 antisence	CTTGTACTCCGTCAGCGTGA
Mouse Notch1 sence	GATGGCCTCAATGGGTACAAG
Mouse Notch1 antisence	TCGTTGTTGTTGATGTCACAGT
CD133 sence	AGTCGGAAACTGGCAGATAGC
CD133 antisence	GGTAGTGTTGTACTGGGCCAAT
NANOG sence	TTTGTGGGCCTGAAGAAAACT
NANOG antisence	AGGGCTGTCCTGAATAAGCAG
CD44 sence	CTGCCGCTTTGCAGGTGTA
CD44 antisence	CATTGTGGGCAAGGTGCTATT
SLUG sence	CGAACTGGACACACATACAGTG
SLUG antisence	CTGAGGATCTCTGGTTGTGGT
PCNA sence	CCTGCTGGGATATTAGCTCCA
PCNA antisence	CAGCGGTAGGTGTCGAAGC
RFC2 sence	GTGAGCAGGCTAGAGGTCTTT
RFC2 antisence	TGAGTTCCAACATGGCATCTTTG
RFC5 sence	GAAGCAGACGCCATGACTCAG
RFC5 antisence	GACCGAACCGAAACCTCGT
Human HES1 sence	TCAACACGACACCGGATAAAC
Human HES1 antisence	GCCGCGAGCTATCTTTCTTCA
Mouse Hes1sence	TCAACACGACACCGGACAAAC
Mouse Hes1antisence	ATGCCGGGAGCTATCTTTCTT
Human HES5 sence	TGCTCAGCCCCAAAGAGAAA
Human HES5 antisence	GAAGGCTTTGCTGTGCTTCA
Mouse Hes5 sence	AGTCCCAAGGAGAAAAACCGA
Mouse Hes5 antisence	GCTGTGTTTCAGGTAGCTGAC
Human HEY1 sence	GTTCGGCTCTAGGTTCCATGT
Human HEY1 antisence	CGTCGGCGCTTCTCAATTATTC
Mouse Hey1 sence	CCGACGAGACCGAATCAATAAC
Mouse Hey1 antisence	TCAGGTGATCCACAGTCATCTG
Human HEY2 sence	AAGGCGTCGGGATCGGATAA

Human HEY2 antisence	AGAGCGTGTGCGTCAAAGTAG
Mouse Hey2 sence	CGCCCTTGTGAGGAAACGA
Mouse Hey2 antisence	CCCAGGGTAATTGTTCTCGCT
Human GAPDH sence	AATGAAGGGGTCATTGATGG
Human GAPDH antisence	AAGGTGAAGGTCGGAGTCAA
Mouse Gapdh sence	AGGTCGGTGTGAACGGATTTG
Mouse Gapdh antisence	GGGGTCGTTGATGGCAACA
