# Supplementary material for: An RFC4/Notch1 signaling feedback loop promotes NSCLC metastasis and stemness
Source: Nat Commun. 2021 May 11;12:2693. doi: 10.1038/s41467-021-22971-x (PMC8113560; doi:10.1038/s41467-021-22971-x)
Supplement: Supplementary file 3 — Description of Additional Supplementary Files [file 41467_2021_22971_MOESM3_ESM.pdf]

Description of Additional Supplementary Files

Supplementary Data 1. NICD1-induced altered genes in A549 cell. The significant altered genes (log2 Fold change > 2 or < -2) in NICD1-overexpressing A549 cell, as compared to their corresponding vector-control cells are listed. Gene expression data were derived from strand-specific sequencing of polyA-enriched RNAs. Data were analyzed by the Wald Chi-Squared Test and adjusted P values were derived by Benjamini-Hochberg method. Original sequencing data have been deposited in GEO database with accession number GSE137106.
