# Supplementary material for: An RFC4/Notch1 signaling feedback loop promotes NSCLC metastasis and stemness
Source: Nat Commun. 2021 May 11;12:2693. doi: 10.1038/s41467-021-22971-x (PMC8113560; doi:10.1038/s41467-021-22971-x)
Supplement: Supplementary file 5 — Reporting Summary [file 41467_2021_22971_MOESM5_ESM.pdf]

nature research

Corresponding author(s): Han Tian, Junchao Cai, Xiuyu Cai

Last updated by author(s): Mar 16, 2021

Reporting Summary

Nature Research wishes to improve the reproducibility of the work that we publish. This form provides structure for consistency and transparency in reporting. For further information on Nature Research policies, see Authors & Referees and the Editorial Policy Checklist.

Statistics

For all statistical analyses, confirm that the following items are present in the figure legend, table legend, main text, or Methods section.

n/a	Confirmed	
☐	☒	The exact sample size (*n*) for each experimental group/condition, given as a discrete number and unit of measurement
☐	☒	A statement on whether measurements were taken from distinct samples or whether the same sample was measured repeatedly
☐	☒	The statistical test(s) used AND whether they are one- or two-sided<br>*Only common tests should be described solely by name; describe more complex techniques in the Methods section.*
☒	☐	A description of all covariates tested
☒	☐	A description of any assumptions or corrections, such as tests of normality and adjustment for multiple comparisons
☐	☒	A full description of the statistical parameters including central tendency (e.g. means) or other basic estimates (e.g. regression coefficient) AND variation (e.g. standard deviation) or associated estimates of uncertainty (e.g. confidence intervals)
☐	☒	For null hypothesis testing, the test statistic (e.g. *F*, *t*, *r*) with confidence intervals, effect sizes, degrees of freedom and *P* value noted<br>*Give P values as exact values whenever suitable.*
☒	☐	For Bayesian analysis, information on the choice of priors and Markov chain Monte Carlo settings
☒	☐	For hierarchical and complex designs, identification of the appropriate level for tests and full reporting of outcomes
☒	☐	Estimates of effect sizes (e.g. Cohen's *d*, Pearson's *r*), indicating how they were calculated

Our web collection on statistics for biologists contains articles on many of the points above.

Software and code

Policy information about availability of computer code

Data collection	FISH images were obtained with a LSM810; confocal microscope using ZEN 2012 software version 8.1 (Carl Zeiss, Oberkochen, Germany); bioluminescent images were taken and analyzed with Spectrum Living Image version 4.2 (Caliper Life Sciences, Waltham, MA, USA);Surface plasmon resonance (SPR) kinetic used BIAevaluate 4.0.1 analysis software.
Data analysis	Western blot grayscale analyses were performed using image J 1.42q software; Statistical analyses were performed using the SPSS 11.0 statistical software package; analysis was done with Graph-Pad Prism 8 version 8.3.0 software (GraphPad software, La Jolla, CA, USA).

For manuscripts utilizing custom algorithms or software that are central to the research but not yet described in published literature, software must be made available to editors/reviewers. We strongly encourage code deposition in a community repository (e.g. GitHub). See the Nature Research guidelines for submitting code & software for further information.

Data

Policy information about availability of data

All manuscripts must include a data availability statement. This statement should provide the following information, where applicable:

- Accession codes, unique identifiers, or web links for publicly available datasets
- A list of figures that have associated raw data
- A description of any restrictions on data availability

The TCGA Lung Adenocarcinoma (LUAD) and Lung Squamous Cell Carcinoma (LUSC) sequencing data used in this study are available in a public repository from the GDC Data Portal Data Release Version 20.0 [https://portal.gdc.cancer.gov/]. The RNA-sequencing data that support the findings of this study has been deposited in GEO with the accession code GSE137106 [https://www.ncbi.nlm.nih.gov/geo/query/acc.cgi?acc=GSE137106]. The source data underlying Figs. 1g, h, j-l, 2a-f, h, i, 3e, 4a-e, h, 5b, d-k, 6a-c, e-g, 7b, e and f, and Supplementary Figs. 1a-c, e, f, h-k, 2a-g, 3a, c-g, i, k, l, 4a-c, f, g, 5a-f, i, 6a-i, 7a-i, 8a, b, d-g, 9a, e and f are provided as a Source Data file. All other data supporting the findings of this study are available from the corresponding author upon reasonable request.

Field-specific reporting

Please select the one below that is the best fit for your research. If you are not sure, read the appropriate sections before making your selection.

☒ Life sciences ☐ Behavioural & social sciences ☐ Ecological, evolutionary & environmental sciences

For a reference copy of the document with all sections, see nature.com/documents/nr-reporting-summary-flat.pdf

Life sciences study design

All studies must disclose on these points even when the disclosure is negative.

Sample size	Sample size was determined by power analysis to achieve a minimum effect size of 0.5 with a P value of less than 0.05 and all sample sizes were appropriate for assumption of normal distribution.
Data exclusions	Clinical specimens histopathologically observed without cancerous lesions and animals bearing tumors in sizes larger than 2cm were excluded from the analysis. The criteria were pre-established.
Replication	For all data presented in the manuscript, , each experiment was successfully repeated at least three times independently under similar condition.
Randomization	A batch of identical cultured cells were randomly allocated to treatment groups and control groups. Animals were randomly put into cages and randomly assigned to experimental groups.
Blinding	For the imaging experiments, where manual counting was required, samples were labeled with numbers in order to avoid preconceptions of the analyzing investigator. For western blot, codification was maintained during sample preparation and decoding was done before loading the samples on the SDS-PAGE gel, in order to ensure an adequate presentation of the results. For other assays, the investigators were also blinded to group allocation during data collection and analysis.

Reporting for specific materials, systems and methods

We require information from authors about some types of materials, experimental systems and methods used in many studies. Here, indicate whether each material, system or method listed is relevant to your study. If you are not sure if a list item applies to your research, read the appropriate section before selecting a response.

Materials & experimental systems

n/a	Involved in the study	
☐	☒	Antibodies
☐	☒	Eukaryotic cell lines
☒	☐	Palaeontology
☐	☒	Animals and other organisms
☐	☒	Human research participants
☒	☐	Clinical data

Methods

n/a	Involved in the study	
☒	☐	ChIP-seq
☐	☒	Flow cytometry
☒	☐	MRI-based neuroimaging

Antibodies

Antibodies used	The antibodies used in western blotting(WB) analysis in the research were: anti-RFC4 (Abcam, Cambridge, MA, ab156780, 1:1000), anti-Notch1 (Cell Signaling, Danvers, MA, 3608, 1:500), anti-NICD1 (Abcam, Cambridge, MA, ab8925, 1:500), anti-p-Ser (Abcam, Cambridge, MA, ab9332, 1:500), anti-p-Thr (Cell Signaling, Danvers, MA, 9381s, 1:500), anti-CDK8 (Abcam, Cambridge, MA, ab224828, 1:1000), anti-FBXW7 (Abcam, Cambridge, MA, ab109617, 1:1000), anti-cyclin C (Abcam, Cambridge, MA, ab85927, 1:1000), anti-RFC2 (Abcam, Cambridge, MA, ab174271, 1:1000), anti-RFC5 (Abcam, Cambridge, MA, ab79871, 1:200), anti-GSK-3β (Abcam, Cambridge, MA, ab32391, 1:1000), anti-MEKK1(Abcam, Cambridge, MA, ab212601, 1:1000), anti-FLAG (Sigma, Saint Louis, MO, USA, F7425, 1:2000), anti-HA (Sigma, Saint Louis, MO, USA, H6908, 1:2000), anti-MYC (Cell Signaling, Danvers, MA, 2278, 1:2000), anti-His antibodies (Abcam, Cambridge, MA, ab9108, 1:2000). Blotted membranes were stripped and re-blotted with anti-p84 (Abcam, Cambridge, MA, ab131268, 1:2000) and anti-β-actin (Sigma, Saint Louis, MO, USA, A2228 1:2000) used as loading controls.
Validation	Antibodies were chosen based on previous literature. Validation and quality control is available from the manufacturers using the catalog number of each antibody. When necessary, additional validations were performed in our laboratory using siRNA- or shRNA- treated cells for the depletion of the targeted protein.<br><br>anti-RFC4 (Abcam, Cambridge, MA, ab156780): mouse monoclonal antibody specific for an epitope mapping between amino acids 1-363, which is recommended for detection of RFC4 of mouse, rat, dog, human and monky by Western Blotting and Immunohistochemistry and Immunofluorescence.

anti-Notch1 (Cell Signaling, Danvers, MA, 3608): Bousquet Mur E,et al. J Clin Invest. 2020;130(2):612-624.

anti-NICD1 (Abcam, Cambridge, MA, ab8925):Hans, C. P.,et al. Sci Rep. 2019;9(1):7999.

anti-p-Ser (Abcam, Cambridge, MA, ab9332): Wang L, et al. Sci Adv. 2020;6(21):eaaz1622.

anti-p-Thr (Cell Signaling, Danvers, MA, 9381s): Lee D, et al. Nat Commun. 2020;11(1):1838.

anti-CDK8 (Abcam, Cambridge, MA, ab224828): Lin Y, et al. Cell Death Dis. 2020;11(1):34.

anti-FBXW7 (Abcam, Cambridge, MA, ab109617):Chen J, et al. Oncogenesis. 2020;9(4):42.

anti-cyclin C (Abcam, Cambridge, MA, ab85927): Xiao li AM, et al. J Biol Chem. 2019;294(34):12743-12753.

anti-RFC2 (Abcam, Cambridge, MA, ab174271): Guenat D, et al. Int J Mol Med. 2017;39(3):622-628.

anti-RFC5 (Abcam, Cambridge, MA, ab79871): Kang MS, et al. Nat Commun. 2019;10(1):2420.

anti-GSK-3β (Abcam, Cambridge, MA, ab32391): Ou B,et al. Cell Death Dis. 2019;10(9):643.

anti-MEKK1(Abcam, Cambridge, MA, ab212601):mouse monoclonal antibody specific for an epitope mapping between amino acids 1077-1076, which is recommended for detection of MEKK1 of human by Western Blotting, Immunohistochemistry, Immunofluorescence and flow cytometry.

anti-FLAG (Sigma-Aldrich, F7425): Shao LW, et al. Nat Commun. 2020;11(1):4639.

anti-HA (Sigma-Aldrich, H3663): Maciaszczyk-Dziubinska E, et al. Nucleic Acids Res. 2020;48(10):5426-5441.

anti-MYC (Cell Signaling, Danvers, MA, 2278): Talwar D, et al. Nat Commun. 2020;11(1):4512.

anti-His (Abcam, Cambridge, MA, ab9108): Jarsch IK, et al. J Cell Biol. 2020;219(4):e201909178.

anti-p84 (Abcam, Cambridge, MA, ab131268): Bachran C, et al. Cell Death Dis. 2014;5(1):e1003.

β-actin (Sigma, Saint Louis, MO, USA, A2228): Patta I, et al. Nucleic Acids Res. 2020;48(11):5873-5890.

Eukaryotic cell lines

Policy information about cell lines

Cell line source(s)	NSCLC cell lines, including A549, H1975, H1703, mouse LLC cell, non-cancerous HEK293FT (293FT) and human umbilical vein endothelial (HUVEC) cells were obtained from the Cell Bank of Shanghai Institutes of Biological Sciences (Shanghai, China) or ATCC, and cultured in DMEM (GIBCO) medium supplemented with 10% fetal bovine serum and 1% penicillin/streptomycin (penicillin 100 U/ml and streptomycin 10 μ g/ml) or LSGS-supplemented Medium 200PRF (for HUVEC, GIBCO). Primary normal lung epithelial (NLE) cells were cultured in Defined Keratinocyte SFM (GIBCO) supplemented with L-glutamine, EGF (20 ng/ml), basic-FGF (10 ng/ml), 2% B27, penicillin/streptomycin and amphotericin B (0.25 mg/ml) 1, 2. obtained according to the protocols of previous reports 3 and cultured in the keratinocyte-serum-free medium (KSFM) as instructed by the provider (GIBCO).
Authentication	All cell lines were authenticated by short tandem repeat (STR) fingerprinting at Medicine Laboratory of Forensic Medicine Department of Sun Yat-Sen University (SYSU) (Guangzhou, China) .
Mycoplasma contamination	All cell lines were tested to be free of mycoplasma contamination.
Commonly misidentified lines (See ICLAC register)	There are no commonly misidentified cell lines were used in the study.

Animals and other organisms

Policy information about studies involving animals; ARRIVE guidelines recommended for reporting animal research

Laboratory animals	BALB/c-nu mice (female, 5-6 weeks of age, 18-20 g) and C57BL/6N (female, 5-6 weeks of age, 18-20 g)
Wild animals	Study did not involve wild animals.
Field-collected samples	Study did not involve field-collected samples.
Ethics oversight	All of the animal procedures were approved by the Sun Yat-sen University Animal Care Committee.

Note that full information on the approval of the study protocol must also be provided in the manuscript.

Human research participants

Policy information about studies involving human research participants

Population characteristics	All clinical tissue specimens used in this study were obtained from and histopathologically diagnosed at the SYSU Cancer Center. Description of population characteristics of the participants: male 163, female 56; age >60yr 86, <60yr 133; Adenocarcinoma 90, Squamous cell carcinoma 74, Adenosquamous carcinoma 17, others 38; stage1 84, stage2 50, stage3 67, stage4 18.
Recruitment	219 NSCLC patients' tumor tissues and adjacent non-tumorous tissues were collected consecutively at the SYSU Cancer Center without artificial selection bias.
Ethics oversight	Prior patients' consent and approval from the Institutional Research Ethics Committee of Sun Yat-sen University were obtained for the use of these clinical materials for research purposes. The study is compliant with all relevant ethical regulations involving human participants.

Note that full information on the approval of the study protocol must also be provided in the manuscript.

Flow Cytometry

Plots

Confirm that:

☒ The axis labels state the marker and fluorochrome used (e.g. CD4-FITC).

☒ The axis scales are clearly visible. Include numbers along axes only for bottom left plot of group (a 'group' is an analysis of identical markers).

☒ All plots are contour plots with outliers or pseudocolor plots.

☒ A numerical value for number of cells or percentage (with statistics) is provided.

Methodology

Sample preparation	For side-population (SP) analysis, indicated cells were dissociated with trypsin and re-suspended at 1×106 cells/ml in DMEM containing 2% fetal bovine serum (FBS) and then incubated at 37°C for 30 min with or without 50 µM Verapamil (Sigma-Aldrich) to inhibit ABC transporters and to confirm the side-population (SP) cells. The cells were subsequently incubated with 5µg/ml Hoechst33342 (Sigma-Aldrich) for 90 min at 37°C, plated on ice for 10 min, washed with ice-cold PBS and subjected to flow cytometry analysis using flow cytometer BD influx (BD Biosciences) as instructed by the manufacturer. For cell cycle analysis, synchronization of cells was facilitated with serum starvation overnight. Cells were fixed in EtOH (70%) over night at 4°C followed by extraction of DNA in DNA-Extraction buffer (0.2 M Na2HPO4, pH 7.8; 0.1% Triton) for 5 min at RT, and staining of DNA in staining-buffer (20µg/ml PI + 200 µg RNaseA) for 15 min at 37°C. Cell cycle phase was checked by flow cytometry analysis using flow cytometer BD influx. And cell apoptosis was evaluated with Annexin V Apoptosis Detection Kit APC (eBioscience).
Instrument	BD influx (BD Biosciences)
Software	BD FACS Sortware
Cell population abundance	Hoechst 33342 is a cell-permeable DNA stain that is excited by ultraviolet light and emits blue fluorescence at 460 to 490 nm. The side-population defines the sub-population of cells with high stemness property.
Gating strategy	Single cells were selected by FSC/SSC, FSC-H/FSC-A and SSC-H/FSC-A gates. Boundaries between “negative” and “positive” staining cell populations were determined by Verapamil treated negative control and fluorescence intensity.

☒ Tick this box to confirm that a figure exemplifying the gating strategy is provided in the Supplementary Information.
